# Exploring the Macroscopic Behavior and Microstructure Evolution of Lightly Cemented Sand in the Post-Liquefaction Process Using DEM

**DOI:** 10.3390/ma17153721

**Published:** 2024-07-27

**Authors:** Fuguang Zhang, Cheng Chen, Huaiping Feng

**Affiliations:** 1 State Key Laboratory of Mechanical Behavior and System Safety of Traffic Engineering Structures, Shijiazhuang Tiedao University, Shijiazhuang 050043, China; fuguang.zhang@stdu.edu.cn; 2 Key Laboratory of Roads and Railway Engineering Safety Control (Shijiazhuang Tiedao University), Ministry of Education, Shijiazhuang 050043, China; 3 School of Civil Engineering, Shijiazhuang Tiedao University, Shijiazhuang 050043, China; 4 Laboratory of Geomechanics and Geotechnical Engineering, Institute of Rock and Soil Mechanics, Chinese Academy of Sciences, Wuhan 430071, China; cchen@whrsm.ac.cn

**Keywords:** cemented sand, post-liquefaction behavior, micromechanism, discrete element modeling

## Abstract

This study investigates the post-liquefaction monotonic undrained shearing behavior of cemented sand at the macro- and microscales, using the discrete element method. A series of cyclic undrained triaxial tests with different stress amplitudes and post-liquefaction monotonic undrained triaxial tests were simulated on cemented sand with diverse cement contents (CCs). For comparison, a series of monotonic undrained triaxial tests on cemented sand without liquefaction (virgin cemented sand) were also modeled. The macroscopic behavior was analyzed in conjunction with the microscopic characteristics of the assembly, such as the deviator fabric of contact normal orientation, mechanical coordination number, energy components, and bond breakage. The results show that the DEM model can capture the effect of CC and cyclic stress ratio (CSR) on the undrained shear strength, stiffness, and pore pressure observed in laboratory experiments. Referring to the virgin specimen, with an increase in CC, the mechanical coordination number and the input work increment increase, while the deviator fabric for total contacts changes irregularly, leading to a greater initial stiffness and shear strength. In the case of the liquefied specimen, the smaller initial mechanical coordination number results in a very low initial stiffness regardless of CC. Contrary to the uncemented sand, both the mechanical coordination number and the input work increment decrease with an increasing CSR for the cemented sand. The microstructure evolution governs the effect of cementation level and liquefaction history on the macroscopic post-liquefaction behavior.

## 1. Introduction

In nature, sand is commonly found with some sort of cementation agent, such as silica, carbonates, iron, and clay minerals [1,2]. In addition, to improve sand response, some ground treatment methods, e.g., cement mixing [3], chemical grouting [4], and microbially induced calcite precipitation [5], among others, have been adopted, leading to the formation of cementation between soil particles. In this paper, such sand possessing inter-particle cementation is termed as cemented sand. It has been recognized that cemented sand is widely distributed in nature and often encountered in engineering practices. An earthquake may cause the liquefaction of cemented sand, and the mechanical response of liquefied cemented sand differs significantly from that without liquefaction. A comprehensive study of post-liquefaction behavior is of great significance to geotechnical engineers, as it permits a safety assessment of structures surviving after an earthquake and a reliability evaluation of soil improvement techniques and guides the design of foundations in liquefaction-prone areas.

The post-liquefaction behavior of cemented sand has been studied using laboratory tests [6,7,8,9,10]. Rasouli et al. (2020) [6], using cyclic and monotonic triaxial tests, investigated the liquefaction and post-liquefaction behavior of lightly cemented sand. The results showed that the post-liquefaction undrained shear strength and initial and secant shear modulus reduced significantly due to liquefaction history. Lee et al. (2022) [9] and Lee and Gomez (2024) [10] studied the post-liquefaction strain accumulation behavior of sand with different cementation levels. The obtained results highlighted that, at higher cementation levels, the cemented sand exhibited distinct reductions in post-liquefaction strain accumulation rates when compared with uncemented sand at similar dry densities, while, at low cementation levels, no improvements were observed in post-liquefaction strain accumulation. However, there is still one question that remains unclear: why does liquefied cemented sand behave differently from virgin cemented sand subjected to the same loading condition? Since the macroscopic mechanical behavior of cemented sand is governed by its microstructure characteristics, answering this question requires further exploration at the microscale.

To characterize the microstructure of cemented sand, scanning electron microscopy [6,7,11,12,13,14], X-ray tomography [15], and computed tomography [16] have been used. Tagliaferri et al. (2011) [15] derived the changes in porosity, cement density, and strain fields within the specimen during monotonic drained triaxial tests. Rasouli et al. (2022) [7] performed multistage liquefaction–re-liquefaction tests and found that most of the bonds broke on or close to the potential shear failure plane, while the sand outside this zone generally remained cemented at the end of tests. However, only the microstructure characteristics after testing were represented in most previous studies. Furthermore, to the best of our knowledge, there are still no studies on the micromechanical behavior of cemented sand during the post-liquefaction process.

The discrete element method (DEM), proposed by Cundall and Strack (1979) [17], can provide the fundamental mechanism underlying the macroscopic response of granular materials. It has been successfully employed to simulate macroscopic post-liquefaction behavior and the particle-scale mechanism of uncemented sand [18,19,20,21,22,23,24,25,26]. For example, Wang et al. (2016) [18] studied the post-liquefaction shear deformation of sand by simulating two-dimensional (2D) cyclic undrained loading and proposed a new fabric entity reflecting the space arrangement of particles. It was found that the new proposed parameter had a strong relationship with post-liquefaction shear strain development. Yang and Huang (2022) [21] conducted 3D DEM simulations of multiple-liquefaction and reconsolidation tests to investigate the effects of soil fabric on liquefaction resistance. The results showed that there was a unified relationship between multiple-liquefaction resistance and soil fabrics, which was independent of strain history and liquefaction stages. Moreover, DEM has also been used to study the mechanical behavior of cemented sand subjected to different loading conditions, such as uniaxial compression [27], isotropic compression [28], monotonic drained triaxial [29,30], and cyclic undrained triaxial tests [31,32]. However, there are still few DEM studies on the micromechanism that underlies the macroscopic post-liquefaction behavior of cemented sand.

This research aims to investigate the micromechanism underlying the post-liquefaction response of cemented sand using 3D DEM simulations. A series of monotonic undrained triaxial tests were modeled on cemented sand with or without cyclic liquefaction history, where different levels of cementation and magnitudes of cyclic loading were considered. The evolution of some important microscale parameters during monotonic undrained shearing was analyzed to study the fundamental mechanism of post-liquefaction behavior.

## 2. DEM Modeling

### 2.1. Bond Contact Model

A bond contact model, accurately describing the effect of inter-particle cementation, is of great importance to the DEM modeling of cemented sand. In this study, a 3D contact model [33] previously developed by our team was adopted. Note that this contact model has been implanted in a DEM code to investigate the cyclic liquefaction behavior of cemented sand [31]. For readability, the contact model will be described concisely in the following.

The contact model mainly consists of two components. The first one is the relationships between the contact forces in normal, tangential, rolling, and twisting directions and the relative displacements corresponding to the contact forces. The second one is the bond failure criterion controlling the state of a bond contact (broken or intact). The contact model defines two typical kinds of bond model, i.e., the thin one and the thick one [34]. In the thin bond model, the bond generates between particles in contact, so the bond contact and the particle contact jointly bear the imposed load. In the thick bond model, the bond forms between particles with an initial gap, and only the bond contact transmits the external load.

**Figure 1:**
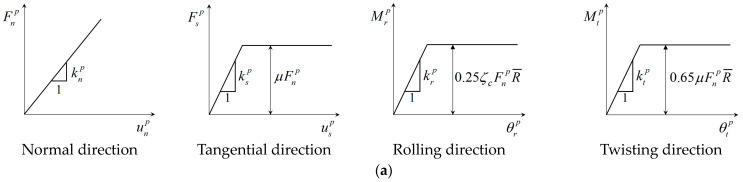

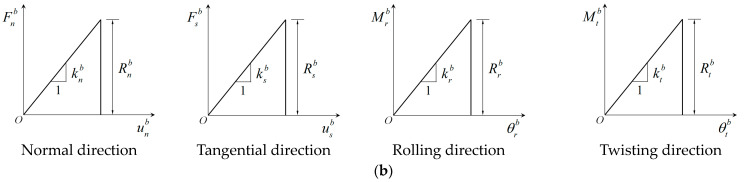
Mechanical response of (**a**) particle contact and (**b**) bond contact [33].

For the thin bond model, the total contact forces can be expressed as follows [33]:

(1) $$F_{n}=F_{n}^{p}+F_{n}^{b}$$

(2) $$F_{s}=F_{s}^{p}+F_{s}^{b}$$

(3) $$M_{r}=M_{r}^{p}+M_{r}^{b}$$

(4) $$M_{t}=M_{t}^{p}+M_{t}^{b}$$

where $F_{n}$, $F_{s}$, $M_{r}$, and $M_{t}$ are the normal, tangential, rolling, and twisting forces, respectively, b and p indicate the bond contact and the particle contact, respectively. Figure 1 shows the diagram of the mechanical response of particle contact and bond contact, where k, μ, $\zeta_{c}$, and $\bar{R}$ are the contact stiffness, frictional coefficient, coefficient of local crushing, and equivalent contact radius, respectively, and $R_{n}^{b}$, $R_{s}^{b}$, $R_{r}^{b}$, and $R_{t}^{b}$ are the normal, tangential, rolling, and twisting resistance of the bond, respectively.

As shown in Figure 1, when any of the contact forces (i.e., normal force, tangential force, rolling moment, and twisting moment) exceed the corresponding bond resistance, the bond contact will fail and cannot transfer any loads. Consequently, the contact model can only consider the rolling and twisting resistances between particles. The failure criterion can be expressed as follows [33]:

(5) $$\left(\frac{F_{s}^{b}}{R_{s}^{b}}\right)+\left(\frac{M_{r}^{b}}{R_{r}^{b}}\right)+\left(\frac{M_{t}^{b}}{R_{t}^{b}}\right)\left\{\begin{array}{c} <1\;\;\mathrm{Intact}\;\;\;\;\\ =1\;\;\mathrm{Critical}\;\\ <1\;\mathrm{Broken}\;\; \end{array}\right.$$

For the thick bond model, the total contact forces can be simplified as those only transferred by the bond contact, since the stiffnesses of the bond contact are smaller than those of the particle contact along different directions. Hence, both the relationships between the contact forces and the relative displacements and the bond failure criterion are similar to those for the thin bond model.

It should be noted that, without considering the actual bond materials, the bond contact model is capable of representing the effect of low levels of cementation due to slight cementation-induced densification. Hence, for simplicity, the low levels of cementation, i.e., 1% and 2%, were considered in this study.

### 2.2. Simulation Procedure

A series of cyclic undrained triaxial tests followed by post-liquefaction monotonic undrained triaxial tests were conducted using the modified DEM code incorporating the aforementioned contact model. In addition, the monotonic undrained triaxial tests without cyclic liquefaction were also simulated to show the effect of liquefaction history on the monotonic undrained shearing behavior of soils. It is worth mentioning that the cyclic undrained shearing behavior of cemented sand has been studied by Zhang et al. (2022) [31].

**Figure 2:**
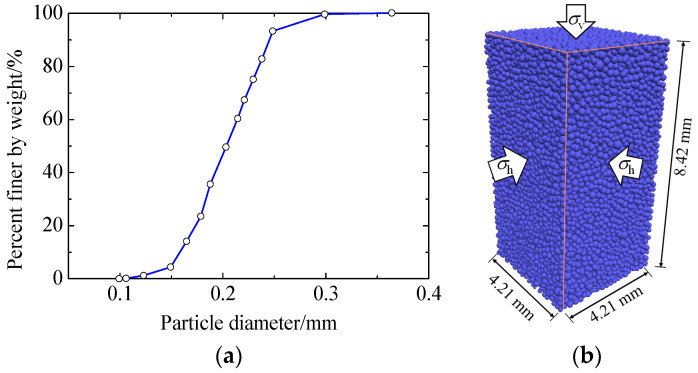
Specimen in DEM simulations: (**a**) particle size distribution of the specimen; (**b**) specimen and applied stresses (walls are omitted).

The mechanical behavior of Toyoura sand has been widely tested all over the world. Meanwhile, the DEM simulations of Toyoura sand with or without cementation have been conducted by our team [33,35]. Hence, Toyoura sand was modeled in this study. To reflect the characteristics of the particle size distribution of Toyoura sand (Figure 2a), a total of 20,000 particles with 15 different sizes were first created using the multilayer under-compaction method [36]. The maximum and minimum void ratios of actual Toyoura sand are 0.972 and 0.609, respectively. To better study the effect of cementation, the resulting loose uncemented specimen had an initial void ratio of 0.80. Then, a confining pressure of 50 kPa was applied to the specimen to simulate the effective stress at a depth of about 5 m. Shen et al. (2016) [33] and Zhang et al. (2022) [31] adopted the same initial stress condition in their DEM studies. Afterward, bonds were generated at the contacts meeting the bond formation criterion [31]. Three different cement contents (CCs), i.e., 0%, 1%, and 2%, were modeled. Certainly, higher levels of cementation, e.g., 10~12%, will also be encountered in practice. Figure 2b shows the prepared specimen. It is worth mentioning that, to study the liquefaction or post-liquefaction behavior of sand using DEM, both cuboid and cylindrical specimens have been widely used in the literature. In this study, a cuboid specimen was employed. Although spherical particles were used to represent sand grains, the rolling and twisting resistances at particle contacts considered in the contact model can effectively represent a real particle shape. This method has been used by many researchers, i.e., Shen et al. (2016) [33] and Zhang et al. (2022) [31], among others. Table 1 shows the input parameters used for particle contacts and bond contacts, which have been determined in [31] in detail.

**Figure 3:**
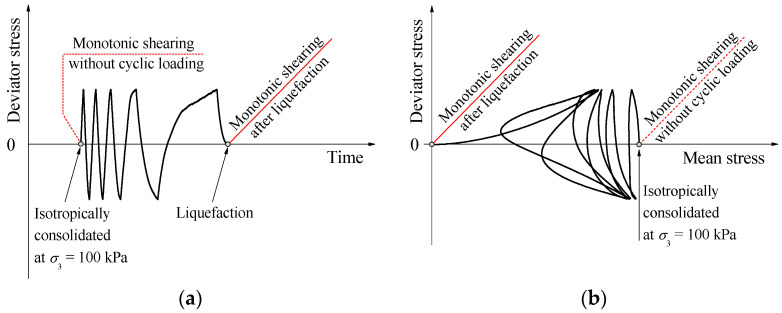
Schematic diagram of tests: (**a**) cyclic loading and monotonic shearing; (**b**) corresponding stress path.

Figure 3 conceptually illustrates the DEM simulation procedure regarding the multistage tests in the current study. After preparation, the specimen with different CCs was first isotropically compressed to a confining pressure of 100 kPa, which has been widely used in the available DEM simulations and laboratory tests. Then, a series of cyclic undrained triaxial tests on the specimen were modeled. In the simulations, the axial load along the vertical direction was applied to the specimen by moving the top and bottom walls toward each other at a strain rate of 6 × 10^−4^/min. Once the deviator stress *(*$q=\sigma_{v}-\sigma_{h}$, where $\sigma_{v}$ and $\sigma_{h}$ are the vertical and horizontal stresses, respectively) arrived at the target stress amplitude, the top and bottom walls moved backward at the same strain rate. To simulate the undrained condition, the positions of the lateral walls were adjusted continuously in accordance with the movements of the top and bottom walls, so that the specimen volume remained constant. The simulation was halted when initial liquefaction occurred for the specimen, at which the pore pressure almost increased up to 100 kPa. Certainly, liquefaction might not occur in cases of low stress amplitudes and high cement contents. Following liquefaction, some liquefied specimens were monotonically sheared under an undrained condition. The specimens were compressed at a strain rate of 2 × 10^−4^/s by moving the top and bottom walls. In addition, in the monotonic undrained triaxial tests without liquefaction history, the loading rate of 2 × 10^−4^/s was also applied to the specimens. Both the loading rates mentioned above met the requirement of the quasi-static condition. A summary of the simulations conducted in this study is presented in Table 2, where the cyclic stress ratio (CSR) is the ratio of the deviator stress to the stress amplitude. These CSRs were selected by referring to previous experimental studies, as they are often used in laboratory tests. The minimum and maximum values of CSRs for a given CC were employed in the modeling of the post-liquefaction monotonic undrained triaxial tests. A workstation with an Intel(R) Core(TM) i7-7700K CPU @ 4.20GHz was used in this study, and the average calculation time of each DEM numerical test was approximately 20 days.

The objective of this study is to investigate the fundamental mechanism underlying the post-liquefaction monotonic undrained shearing behavior of cemented sand. Hence, this study does not aim to reproduce the mechanical response of any specific cemented sand from laboratory tests.

## 3. DEM Simulation Results

### 3.1. Macrocyclic Liquefaction Behavior

In the past study performed by Zhang et al. (2022) [31], the cyclic liquefaction response of cemented sand with different CCs under diverse CSRs was studied in detail. For readability, some obtained results will be presented.

**Figure 4:**
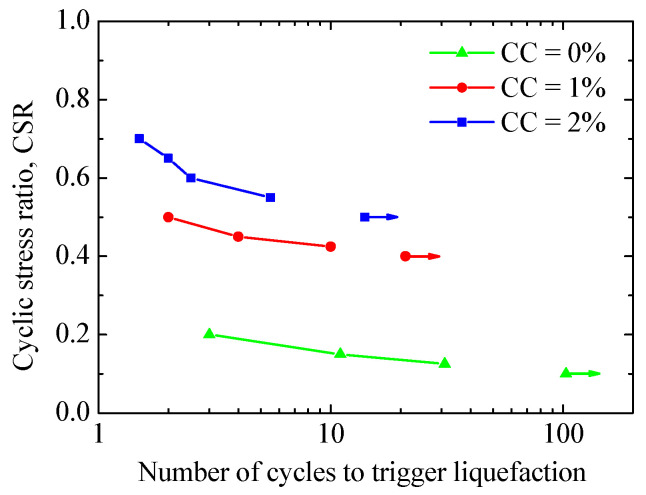
Effect of cementation on liquefaction resistance (modified from [31]).

**Figure 5:**
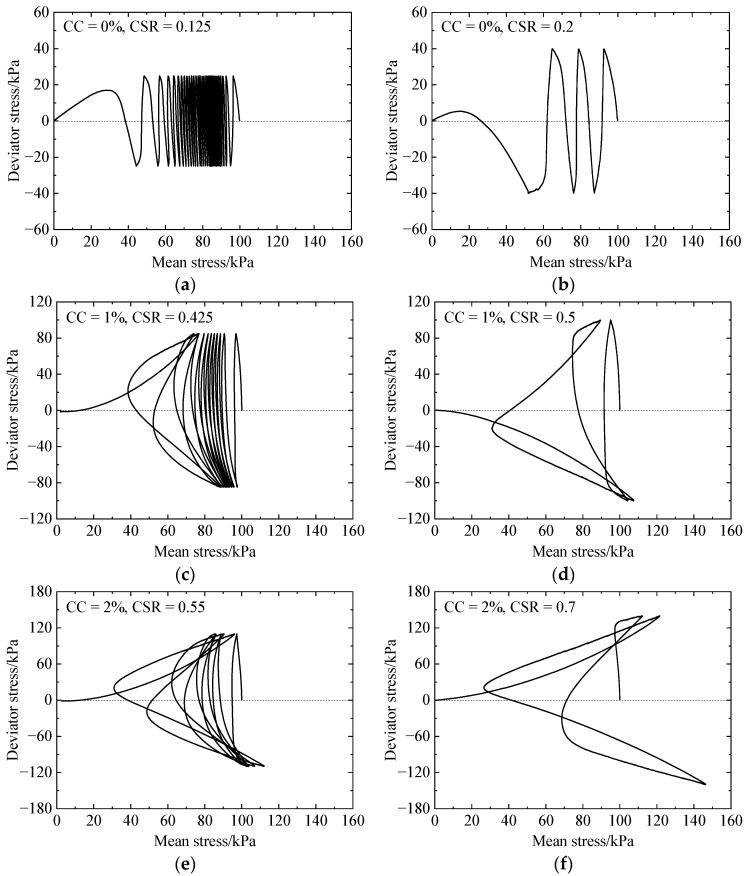
Stress paths for different specimens under cyclic undrained shearing with variable CSRs: (**a**) CC = 0%, CSR = 0.125; (**b**) CC = 0%, CSR = 0.2; (**c**) CC = 1%, CSR = 0.425; (**d**) CC = 1%, CSR = 0.5; (**e**) CC = 2%, CSR = 0.55; (**f**) CC = 2%, CSR = 0.7.

Figure 4 presents the liquefaction resistance curves (CSRs versus number of cycles to liquefaction) for different CCs and CSRs, where the arrow pointing to the right indicates no occurrence of liquefaction at that number of loading cycles. The figure shows that the liquefaction resistance increases with increasing CCs or decreasing CSRs, and the curve exhibits an exponential behavior, which are in accordance with the experimental results [6,14,37]. Figure 5 provides typical stress paths (deviator stress versus mean stress) under cyclic loading, including CC = 0% under CSR = 0.125 and 0.2, CC = 1% under CSR = 0.425 and 0.5, and CC = 2% under CSR = 0.55 and 0.7. As shown in Figure 5, both the deviator and mean stresses gradually approach zero, meaning that initial liquefaction eventually occurs for all the specimens.

### 3.2. Macro Post-Liquefaction Response without Liquefaction

For comparison, the monotonic undrained shearing behavior of sand with different CCs without liquefaction history is first presented.

**Figure 6:**
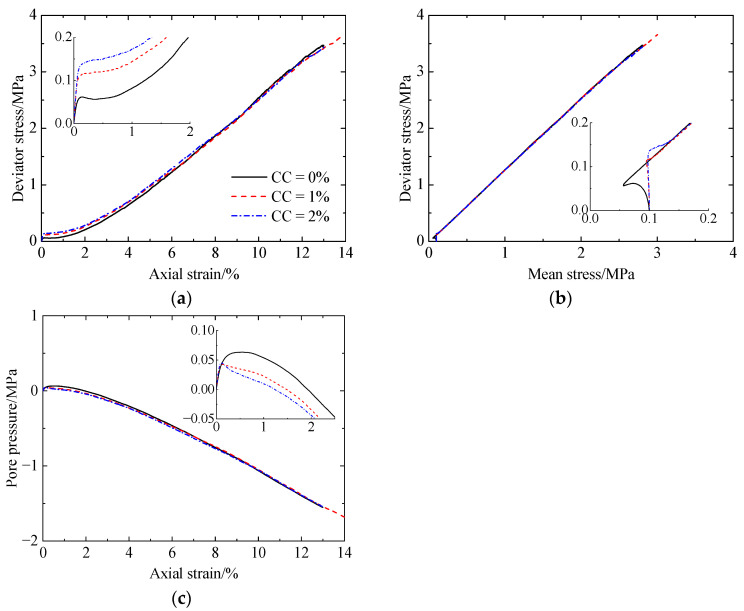
Monotonic undrained shearing behavior of the specimens without liquefaction: (**a**) deviator stress versus axial strain; (**b**) deviator stress versus mean stress; (**c**) pore pressure versus axial strain.

Figure 6 shows the corresponding simulation results, including deviator stress versus axial strain (Figure 6a), deviator stress versus mean stress (Figure 6b), and pore pressure versus axial strain (Figure 6c). As shown in Figure 6a, the deviator stress for the uncemented sand (CC = 0%) abruptly increases to a peak value at a strain of 0.1% and then slightly decreases at strains smaller than 0.5%. Subsequently, the deviator stress increases significantly. For the cemented sand (CC = 1% and 2%), the deviator stress increases abruptly at strains smaller than 0.1% and then increases at a very low rate at strains smaller than 0.5%. Afterward, the deviator stress increases rapidly. As the CC increases, the increase in the mean stress (Figure 6b) results in the increases in the shear strength and stiffness (Figure 6a). The specimens all possess identical shear strengths at strains larger than 8%. Further, no differences in the failure envelopes can be observed with increasing cementation levels (Figure 6b). As shown in Figure 6c, all the specimens exhibit initially contractive tendencies at strains smaller than 1.25%. An increasingly dilative volumetric tendency can be observed at larger strains with an increase in cementation levels; once the strain exceeds 8%, the specimens exhibit almost the same dilative behavior, since the same negative pore pressure generates regardless of CCs. The effect of cementation on the shearing behavior described above is in agreement with the experimental data of Lee et al. (2022) [9]. In [9], a series of monotonic undrained triaxial tests were conducted on lightly biocemented loose Ottawa F-65 sand with CaCO_3_ contents of 0.32%, 0.40%, 0.64%, and 0.71% by mass. Compared with the experimental results [9], a greater initial stiffness and shear strength at a given strain can be observed in the simulation results. These differences are mainly attributed to the greater strength of cementation and higher density of the specimen used in this study. Note that all the specimens do not reach critical states even at larger strains, which is attributed to the high relative density.

### 3.3. Macro Post-Liquefaction Response after Liquefaction

**Figure 7:**
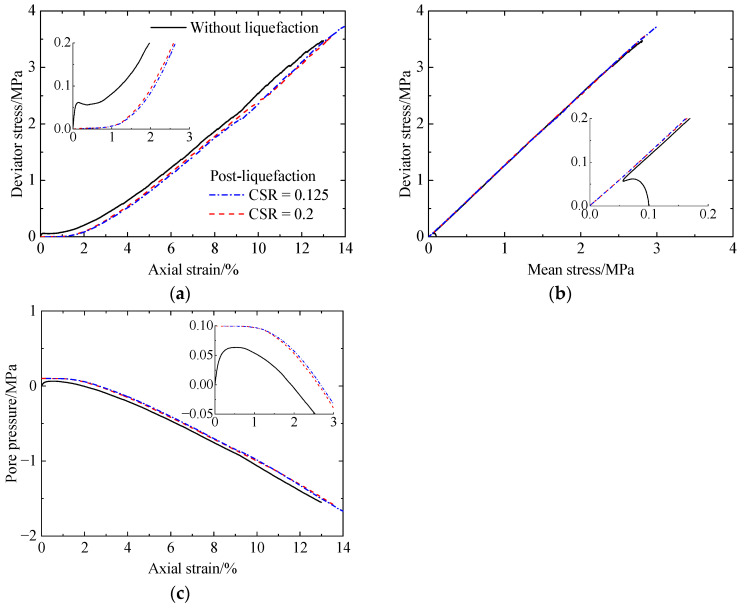
Monotonic undrained shearing behavior of the uncemented sand after liquefaction under CSRs of 0.125 and 0.2: (**a**) deviator stress versus axial strain; (**b**) deviator stress versus mean stress; (**c**) pore pressure versus axial strain.

**Figure 8:**
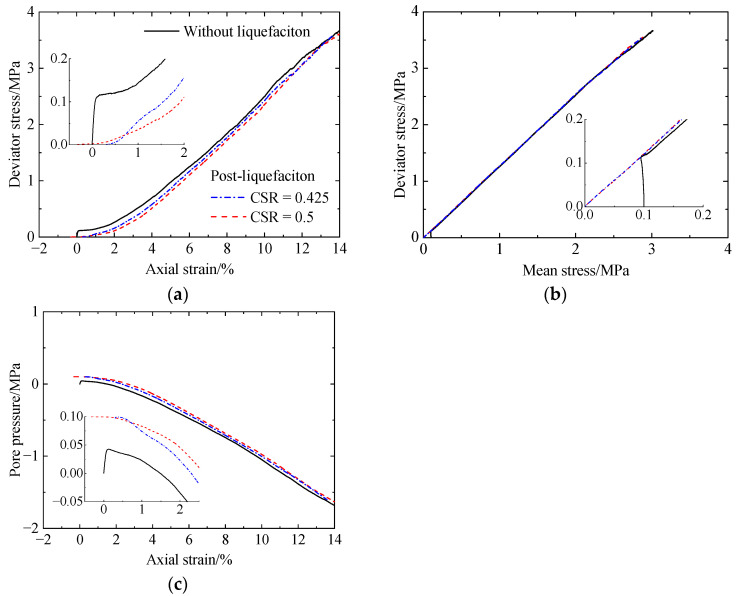
Monotonic undrained shearing behavior of the cemented sand with CC = 1% after liquefaction under CSRs of 0.425 and 0.5: (**a**) deviator stress versus axial strain; (**b**) deviator stress versus mean stress; (**c**) pore pressure versus axial strain.

**Figure 9:**
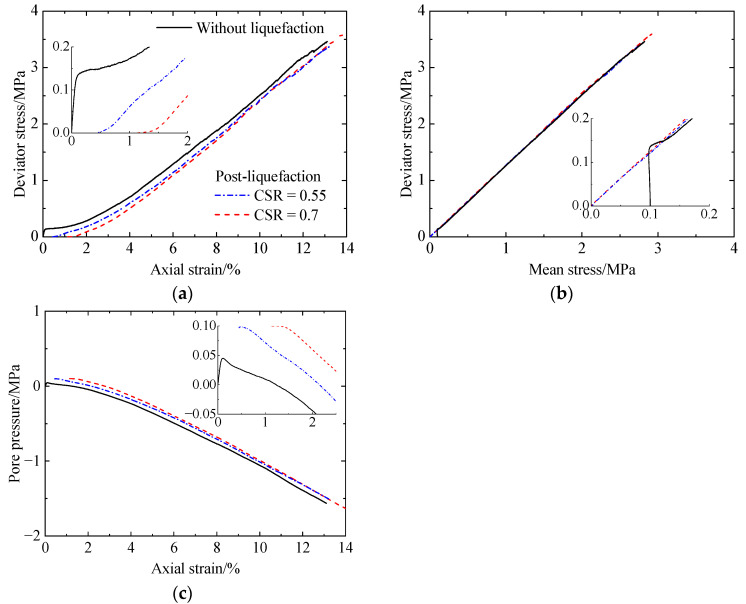
Monotonic undrained shearing behavior of the cemented sand with CC = 2% after liquefaction under CSRs of 0.55 and 0.7: (**a**) deviator stress versus axial strain; (**b**) deviator stress versus mean stress; (**c**) pore pressure versus axial strain.

Figure 7, Figure 8 and Figure 9 present the post-liquefaction monotonic undrained shearing behavior of sand with a CC of 0%, 1%, and 2% after liquefaction under various CSRs, respectively, which include deviator stress versus axial strain, deviator stress versus mean stress, and pore pressure versus axial strain. For comparison, the corresponding results without liquefaction are also included. For uncemented sand after liquefaction (Figure 7), it shows a very low initial stiffness (Figure 7a), which has been stated by other researchers [6,38]. Note that the used materials differed between the experiments [6,38] and this study. When the axial strain exceeds 1%, the considerable decrease in pore pressure (Figure 7c) suppresses volumetric dilation, leading to a significant increase in stiffness. As the CSR increases from 0.125 to 0.2, pore pressure decreases, while shear strength increases (Figure 7a). The shear strength of the specimen after liquefaction is lower than that without liquefaction. Even so, there are no differences between the failure envelopes for the specimens with or without liquefaction (Figure 7b). Referring to the cemented sand with CC = 1% (Figure 8), the initial stiffness after liquefaction is also relatively low compared to that without liquefaction (Figure 8a). As the CSR increases from 0.425 to 0.5, the pore pressure increases (Figure 8c), leading to reductions in the initial stiffness and shear strength (Figure 8a). In addition, as the axial strain increases, the effect of prior liquefaction on the shear strength becomes less distinguished, which is the same as that for the pore pressure. Likewise, the specimens all exhibit the same failure envelopes (Figure 8b). Increasing the CC from 1% to 2% (Figure 9), the variation in stiffness, shear strength, and pore pressure with prior liquefaction and cementation remains unchanged. The effect of cementation and liquefaction history on the shearing behavior is in accordance with the experimental data of Rasouli et al. (2020) [6]. Note that all the numerical specimens after liquefaction undergo strain hardening, and the pore pressure always decreases within the strains studied. In the experiments [6], the specimens with low cementation levels almost reach the peak strength, and the pore pressure decreases slowly at a strain of 14%. For the specimens with higher cementation levels, they experience strain softening after reaching the peak strength. These differences are mainly attributed to the higher density of the specimens used in this study.

### 3.4. Description of Micromechanical Parameters

The above analysis indicates that the current DEM model can effectively capture the monotonic undrained shearing behavior of liquefied and virgin sand with different CCs. Apart from the analysis of the macroscopic behavior, the DEM also allows an examination of the evolution of microstructure represented by several microscopic quantities (i.e., bond breakage, deviator fabric, mechanical coordination number, and energy components).

The macromechanical behavior of cemented sand is strongly governed by bond breakage events. According to the loading condition where a bond breaks, bond breakage can be divided into two types, i.e., breakage due to tension and breakage due to compression. For bond breakage due to tension, it occurs when the tensile force or a combined force under tension exceed the tensile resistance of the bond. When the compressive force or a combined force under compression exceed the compressive resistance of the bond, the bond breaks due to compression.

Fabric tensor, proposed by Sataka (1982) [39], has been widely used to represent the anisotropy of a granular system. The fabric tensor $F_{ij}$ can be expressed as follows:

(6) $$F_{ij}=\frac{1}{N_{c}}\sum_{N_{c}}n_{i}n_{j}$$

where $N_{i}$ and $N_{j}$ are the contact normal orientations of the *i*- and *j*-directions, respectively (*i*, *j* = 1, 2, and 3).

A scalar parameter $F_{d}$ was used to quantify the degree of fabric anisotropy [40]:

(7) $$F_{d}=\sqrt{\left[{\left(F_{1}-F_{2}\right)}^{2}+{\left(F_{1}-F_{3}\right)}^{2}+{\left(F_{2}-F_{3}\right)}^{2}\right]/2}$$

where $F_{1}$,$F_{2}$, and $F_{3}$ are the maximum, intermediate, and minimum principal fabrics, respectively. Using the same numerical specimens as those in the current study, Zhang et al. (2022) [31] confirmed that the direction of the principal fabric for different types of contacts, i.e., bonded contacts, unbonded contacts, broken bond contacts, and total contacts, may be horizontal or vertical during loading. Therefore, the deviator fabric can be calculated as $F_{d}=F_{1}-F_{2}=F_{z}-F_{x}$. A positive $F_{d}$ indicates the preferred contact normal orientation of the vertical direction.

Following Thornton (2000) [41], the mechanical coordination number was used to represent the effect of contact density:

(8) $$Z_{m}=\frac{2N_{c}-N_{p}^{1}}{N_{p}-N_{p}^{0}-N_{p}^{1}}$$

where $N_{c}$ and $N_{p}$ are the numbers of the contacts and the particles, respectively, and $N_{p}^{0}$ and $N_{p}^{1}$ are the numbers of particles with 0 or 1 contact, respectively. In the current study, the unbonded and bonded contacts in the cemented sand were both considered.

Energy storage and dissipation are closely related to the macromechanical response of granular materials subjected to loading. Hence, the evolution of different energy components, including the elastic strain energy stored at particle and bond contacts, plastic energy dissipated at particle contacts due to frictional sliding, rolling rotation, and twisting rotation, will be studied. The elastic energy stored at particle contacts $E_{e}^{p}$ and its increment ${∆E}_{e}^{p}$ are calculated as follows:

(9) $$E_{e}^{p}=\sum_{N_{p}}\frac{{\left(F^{p}\right)}^{2}}{2k^{p}}$$

(10) $${∆E}_{e}^{p}=E_{e}^{p(t+∆t)}-E_{e}^{p(t)}$$

where $F^{p}$ is the contact force or moment, $k^{p}$ is the corresponding contact stiffness, and ∆t is the time step.

Similarly, the elastic energy stored at bond contacts and its increment can be derived from Equations (9) and (10), provided that the force, moment, and stiffness for the particle contacts are replaced with those for the bond contacts. In addition, the plastic energy dissipated at particle contacts $E_{p}^{p}$ and its increment ${∆E}_{p}^{p}$ are calculated as follows:

(11) $$E_{p}^{p}\leftarrow E_{p}^{p}-\sum_{N_{p}}F^{p}{∆d}_{p}$$

(12) $${∆E}_{p}^{p}=\sum_{N_{p}}F^{p}{∆d}_{p}$$

where ${∆d}_{p}$ is the increments of sliding displacement, rolling rotation, and twisting rotation.

### 3.5. Micromechanical Response without Liquefaction

Before describing the micromechanical behavior of the cemented liquefied sand, the corresponding results for the virgin sand will be introduced first.

**Figure 10:**
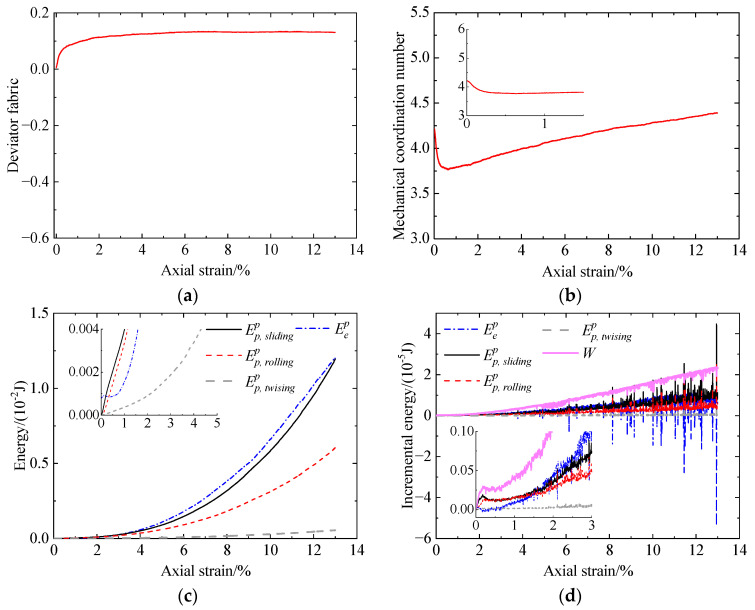
Microscopic monotonic undrained shearing behavior of the uncemented sand without liquefaction: (**a**) deviator fabric versus axial strain; (**b**) mechanical coordination number versus axial strain; (**c**) energy versus axial strain; (**d**) incremental energy versus axial strain.

Figure 10 shows the microscopic behavior of the uncemented sand without liquefaction history, including deviator fabric versus axial strain (Figure 10a), mechanical coordination number ($Z_{m}$) versus axial strain (Figure 10b), and energy versus axial strain (Figure 10c,d). As shown in Figure 10a, the deviator fabric increases considerably from zero at the initial stage and then increases at a decreasing rate to a constant value. This indicates that the contact normal orientations initially are isotropic due to prior isotropic compression and gradually exhibit preferably in the vertical direction to form the force chains along the vertical direction transferring or resisting the vertical load. As shown in Figure 10b, $Z_{m}$ drops abruptly at the initial stage because of the loss of contacts in the horizontal direction [42]. The termination of the decline in $Z_{m}$ corresponds to the initialization of the phase transformation state (Figure 6). Subsequently, $Z_{m}$ gradually increases due to particle rearrangement, resulting in the increase in deviator stress. Figure 10c shows that the input work is mainly dissipated, where the frictional energy is the largest, and the twisting energy is the smallest. The remaining work is stored as elastic energy at particle contacts. Figure 10d shows that the variation in the input work increment (*W*) with axial strain is similar to that of the deviator stress (Figure 6a). The decrease in sliding and rolling energy increments after reaching peak values contributes to that of the deviator stress. As loading further proceeds, the sliding, rolling, and elastic energy increments all increase, among which the ratio of the elastic energy increment increases, leading to the increase in the deviator stress (Figure 6a).

**Figure 11:**
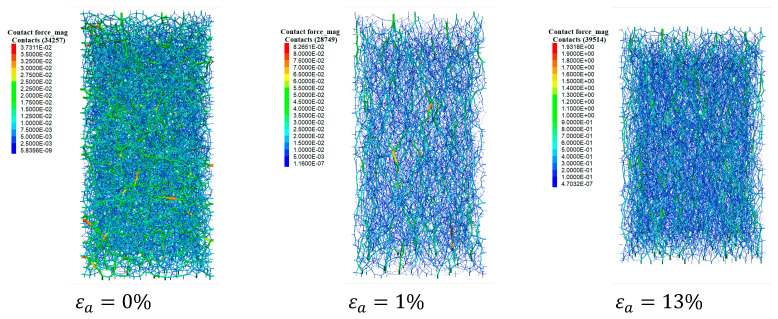
Variation in the force chain network within the uncemented sand at different loading stages.

To observe the evolution of the microstructure intuitively, the force chain network at typical loading stages is shown in Figure 11, including $\varepsilon_{a}=0\%$, $\varepsilon_{a}=1\%$, and $\varepsilon_{a}=13\%$ ($\varepsilon_{a}$ is axial strain). Before loading, the distribution of force chains is isotopic without a preferred orientation. At an axial strain of 1%, it can be found that the force chains exhibit preferably in the vertical direction, and the number of contacts is smaller than that at $\varepsilon_{a}=0\%$. As loading further proceeds, the force chains still indicate the dominance of particle contacts along the vertical direction, and the contact number is larger compared to that at $\varepsilon_{a}=1\%$. These observations confirm the variation in the deviator fabric and $Z_{m}$ obtained from Figure 10a,b.

**Figure 12:**
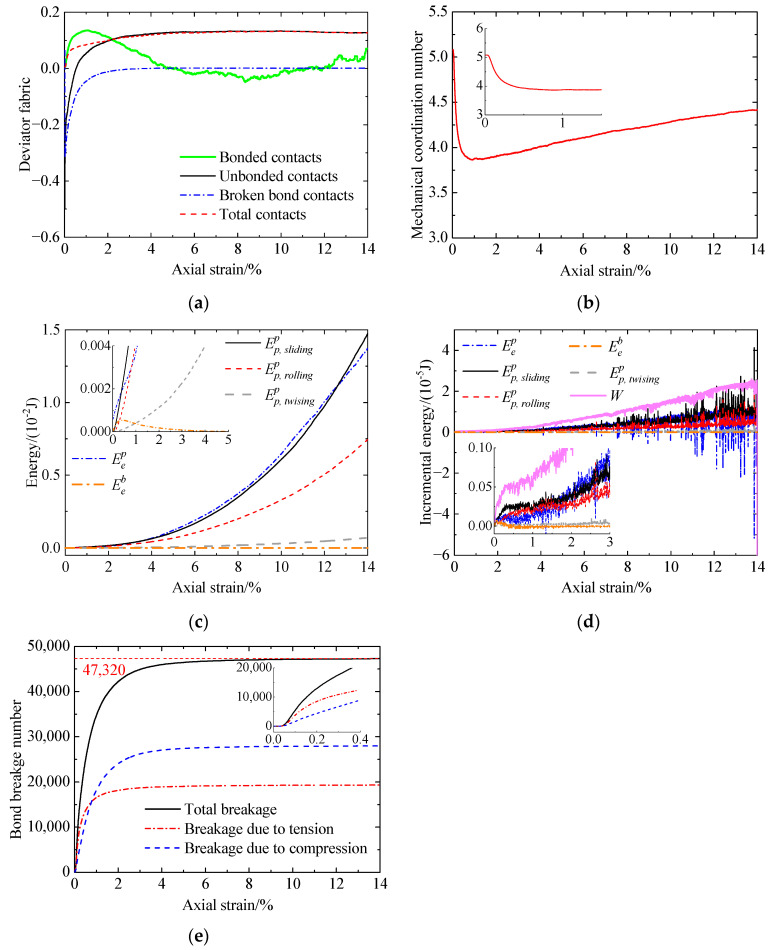
Microscopic monotonic undrained shearing behavior of the cemented sand with CC = 1% without liquefaction: (**a**) deviator fabric versus axial strain; (**b**) mechanical coordination number versus axial strain; (**c**) energy versus axial strain; (**d**) incremental energy versus axial strain; (**e**) bond breakage versus axial strain.

Figure 12 presents the microscopic behavior of the cemented sand with CC = 1% without liquefaction, including deviator fabric versus axial strain (Figure 12a), mechanical coordination number ($Z_{m}$) versus axial strain (Figure 12b), energy versus axial strain (Figure 12c,d), and bond breakage number versus axial strain (Figure 12e). As shown in Figure 12a, the initial deviator fabric with $F_{d}=0$ for the bonded contacts indicates the isotropic behavior of the contact normal orientations due to prior isotropic compression. At the initial stage, a large amount of bond breakage occurs mainly in the horizontal direction due to tension (Figure 12a,e). As a result, the preferred contact normal orientations for the bonded contacts turn to the vertical direction. Afterward, bond breakage mainly due to compression in the vertical direction (Figure 12e) leads to a reduction in the preferred orientation of the vertical direction for the bonded contacts (Figure 12a). As the axial strain exceeds 5%, the deviator fabric for the bonded contacts fluctuates around zero due to very few intact bonds (Figure 12a,e). Referring to the unbonded contacts, they initially exhibit preferably in the horizontal direction due to bond breakage in the same direction. With an increase in bond breakage, the unbonded contacts in the vertical direction ($F_{d}>0$) gradually transfer loading. Due to the rearrangement of the normal orientations of the bonded and unbonded contacts, the preferred direction for total contacts turns vertical to resist loading. For $Z_{m}$ (Figure 12b), it initially remains unchanged mainly due to the occurrence of no bond breakage (Figure 12e). Afterward, bond breakage increases considerably. Meanwhile, the unbonded contacts in the horizontal direction decline. Consequently, $Z_{m}$ decreases sharply. After reduction, $Z_{m}$ gradually increases with increasing axial strain. The smallest $Z_{m}$ occurs at an axial strain of 1%, which is larger than the phase transformation state (Figure 6). This may be because, despite increasing mean stress, the decrease in contacts due to bond breakage overwhelms the increase in contacts due to compaction. Figure 12c illustrates that, with increasing axial strain, the elastic energy at bond contacts increases to a peak at an axial strain of 0.4% and then gradually reduces to zero at strains smaller than 4%. The reason will be explained as follows. At the initial stage of loading, the increase in the force transferred by the bond contacts leads to an increase in the elastic energy. As loading further proceeds, bond breakage occurs significantly, which has an adverse effect on the elastic energy. When the adverse effect outweighs the beneficial effect brought by the contact force increment, the elastic energy will reduce. Furthermore, the input work is mainly dissipated rather than stored at contacts, which is similar to the uncemented sand (Figure 10c). Figure 12d shows the input work increment increases rapidly first and then increases at a decreasing rate. Afterward, the input work increment increases sharply. It can be observed that the variation in the input work increment also agrees well with that of the deviator stress (Figure 6a). The increases in the sliding and rolling energy increments together with the incremental elastic energy stored at particle contacts contribute to that of the deviator stress.

**Figure 13:**
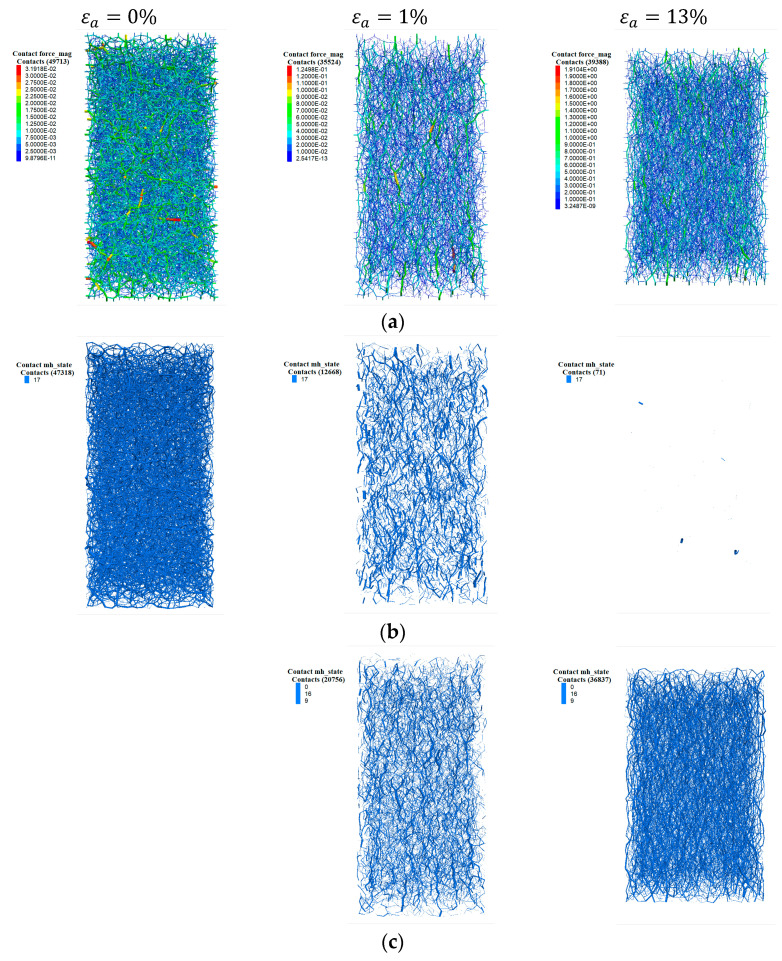
Variation in the force chain network within the cemented sand with CC = 1% at different loading stages: (**a**) total contacts; (**b**) bonded contacts; (**c**) unbonded contacts.

The variation in the force chain network within the cemented sand with CC = 1% at different loading stages is shown in Figure 13, including total contacts (Figure 13a), bonded contacts (Figure 13b), and unbonded contacts (Figure 13c). Likewise, these observations confirm the variation in the deviator fabric for the different types of contacts and the $Z_{m}$.

**Figure 14:**
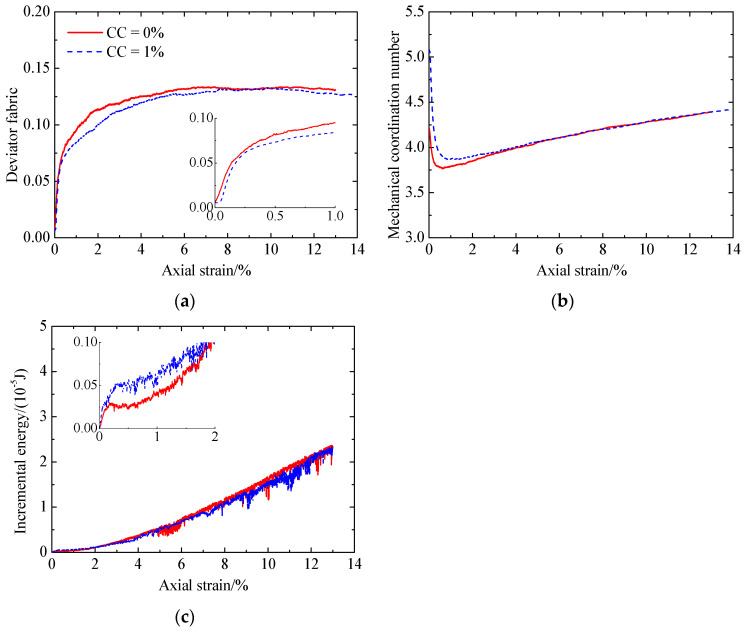
Comparison of the microscopic monotonic undrained shearing behavior of sand with CC = 0% and 1% without liquefaction: (**a**) deviator fabric for total contacts versus axial strain; (**b**) mechanical coordination number versus axial strain; (**c**) input work increment versus axial strain.

Compared to the uncemented sand and the cemented sand with CC = 1% (Figure 14), it can be found that, due to the adding of cementation between particles, the mechanical coordination number increases (Figure 14b), while the deviator fabric for total contacts decreases at smaller strains (Figure 14a). As loading further proceeds, a large amount of bond breakage (Figure 12e) reduces the effect of cementation, leading to almost the same two microscopic quantities for the uncemented and cemented sand (Figure 14a,b). Note that the deviator fabric for total contacts in the case of the cemented sand considers the uncemented and cemented contacts, which exhibit a different response. Whether the deviator fabric for total contacts is able to indicate the shear strength of cemented sand needs further verification. As shown in Figure 14c, the effect of cementation on the input work increment is the same as that for the deviator stress (Figure 6a). With an increase in CC, the increases in the sliding and rolling energy increments as well as the incremental elastic energy stored at particle contacts lead to an increase in the deviator stress (Figure 6a).

**Figure 15:**
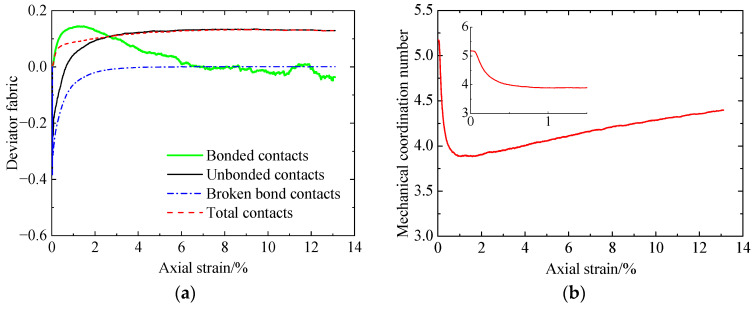

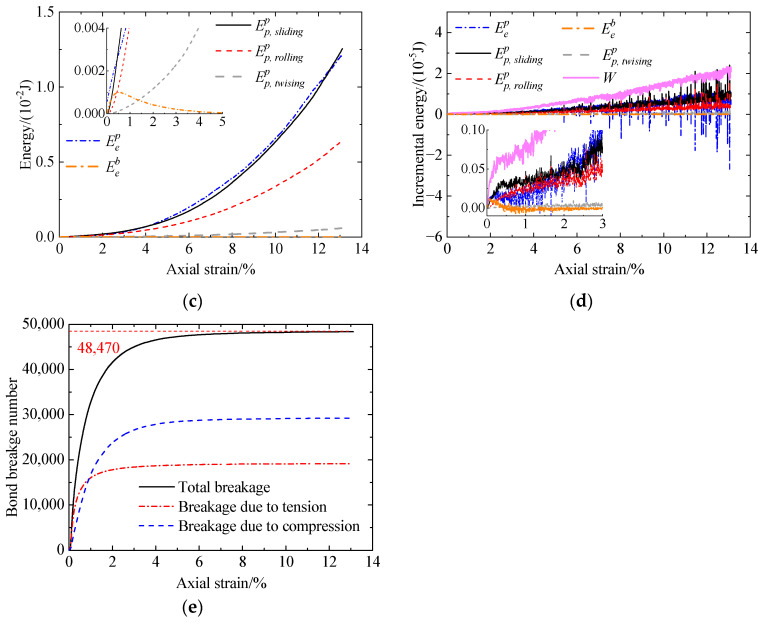
Microscopic monotonic undrained shearing behavior of the cemented sand with CC = 2% without liquefaction: (**a**) deviator fabric versus axial strain; (**b**) mechanical coordination number versus axial strain; (**c**) energy versus axial strain; (**d**) incremental energy versus axial strain; (**e**) bond breakage versus axial strain.

**Figure 16:**
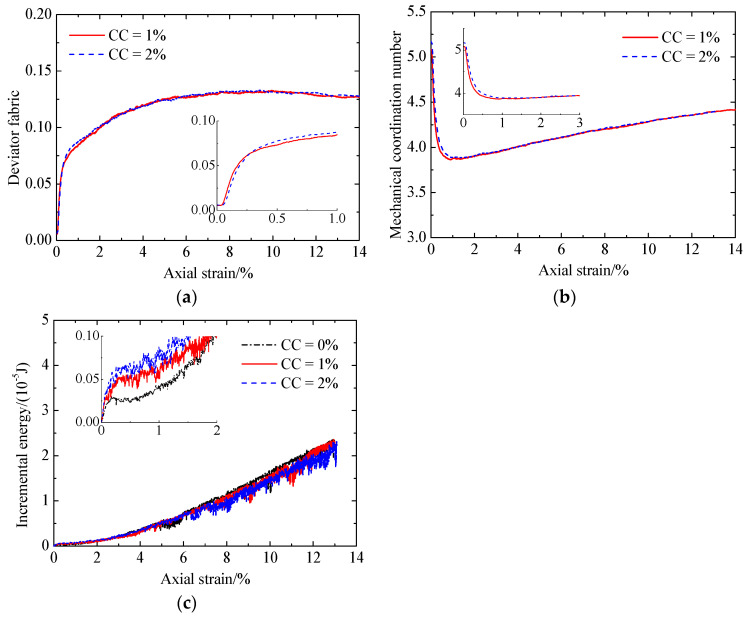
Comparison of the microscopic monotonic undrained shearing behavior of the sand with CC = 1% and 2% without liquefaction: (**a**) deviator fabric for total contacts versus axial strain; (**b**) mechanical coordination number versus axial strain; (**c**) input work increment versus axial strain.

Increasing the CC from 1% to 2% (Figure 15), the evolution of the microscopic quantities is almost the same. With increasing CC, the bond number prior to shearing increases (Figure 12e and Figure 15e). Meanwhile, the mechanical coordination number and the input work increment increase at smaller strains (Figure 16b,c). As a result, the increase in the CC improves the shear strength (Figure 6). In addition, with increasing CC, the deviator fabric for total contacts increases at the very beginning and then decreases, followed by an increase at strains smaller than 2% (Figure 16a). There is no relationship between the effect of cementation on the deviator fabric (Figure 16a) and that on the shear strength (Figure 6a).

### 3.6. Micromechanical Response after Liquefaction

**Figure 17:**
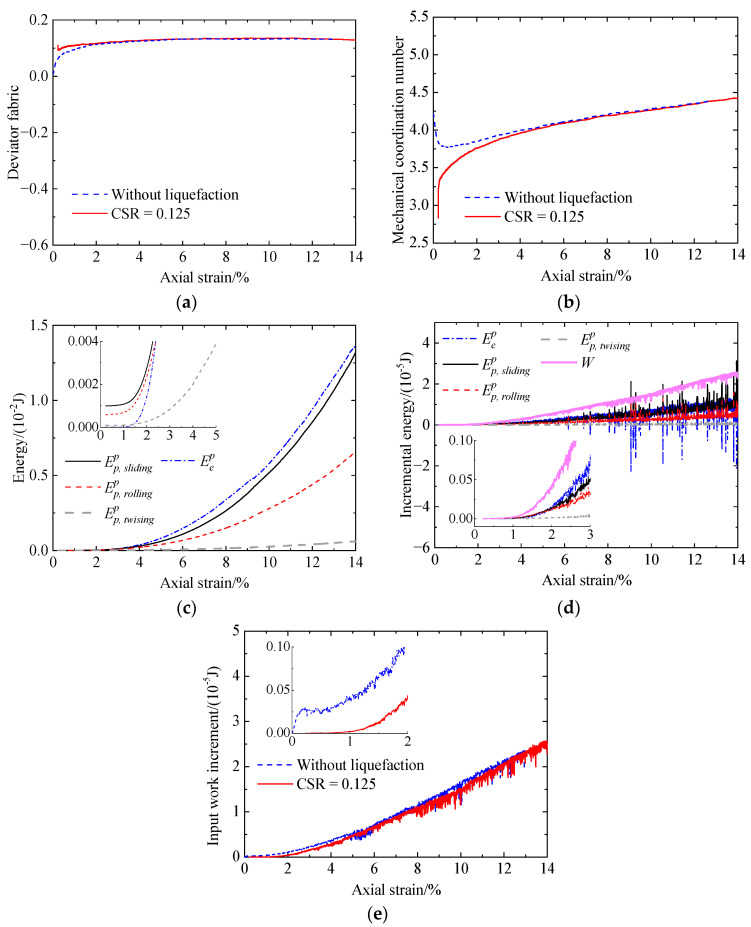
Microscopic monotonic undrained shearing behavior of the uncemented sand after liquefaction under a CSR of 0.125: (**a**) deviator fabric versus axial strain; (**b**) mechanical coordination number versus axial strain; (**c**) energy versus axial strain; (**d**) incremental energy versus axial strain; (**e**) input work increment versus axial strain.

Figure 17 presents the microscopic behavior of the uncemented sand after liquefaction under a CSR of 0.125, including deviator fabric versus axial strain (Figure 17a), mechanical coordination number versus axial strain (Figure 17b), and energy versus axial strain (Figure 17c,d). For comparison, the results without liquefaction history are also included. At the very beginning, both the deviator fabric and the mechanical coordination number change slightly due to the change in loading mode from cyclic shearing to monotonic shearing. As shown in Figure 17a, the initial deviator fabric is about 0.1, indicating a preferred contact normal orientation in the vertical direction. After a slight increase, the deviator fabric reaches a steady value. The deviator fabric for the specimen after liquefaction is larger than that without liquefaction, and the difference decreases with loading. As illustrated in Figure 17b, the mechanical coordination number is very small ($Z_{m}<3$), leading to low stiffness (Figure 7). Further, it increases nonlinearly and is smaller than that without liquefaction, with the difference reducing with further loading. Figure 17d shows that all the incremental energy components do not increase at strains smaller than 1% and then increase sharply. Furthermore, the input work increment decreases due to liquefaction history (Figure 17e). Hence, the specimen with liquefaction has a smaller shear strength.

**Figure 18:**
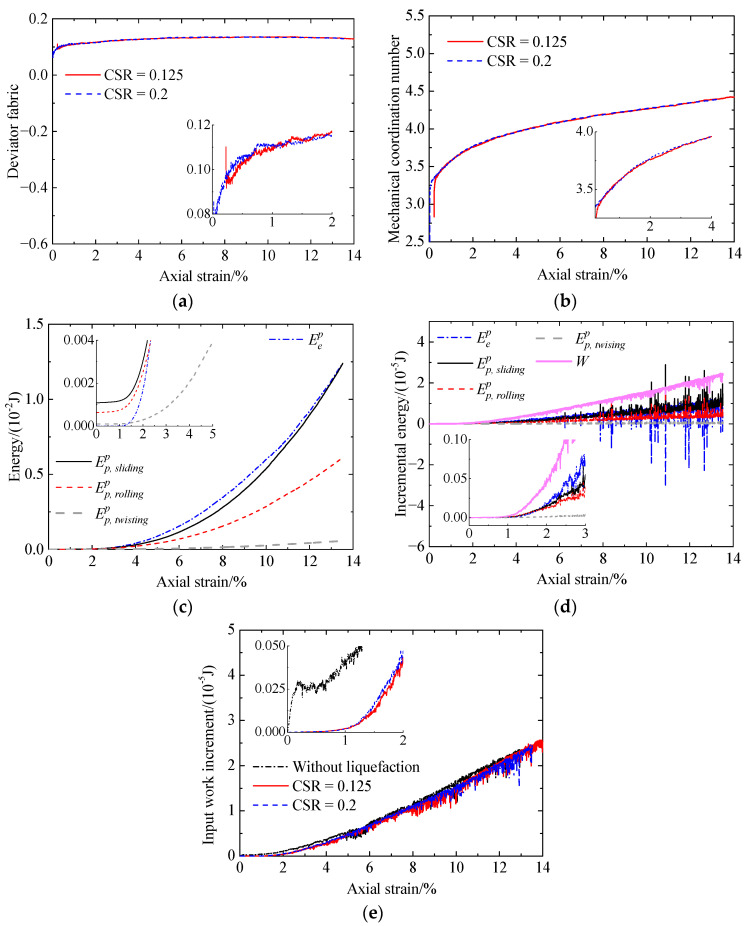
Microscopic monotonic undrained shearing behavior of the uncemented sand after liquefaction under a CSR of 0.2: (**a**) deviator fabric versus axial strain; (**b**) mechanical coordination number versus axial strain; (**c**) energy versus axial strain; (**d**) incremental energy versus axial strain; (**e**) input work increment versus axial strain.

Increasing the CSR from 0.125 to 0.2 (Figure 18), it can be observed that the mechanical coordination number (Figure 18b) and the input work increment (Figure 18e) increase slightly. However, there is no correlation between the deviator fabric (Figure 18a) and the shear strength (Figure 7a) for a CSR of 0.125 and 0.2, as the deviator fabric under a CSR of 0.2 is larger or smaller than that under a CSR of 0.125. From the above analysis, it can be found that the deviator fabric is not a good indicator of the shear strength. Hence, the evolution of the other three parameters, i.e., the mechanical coordination number, incremental energy, and bond breakage, are presented.

**Figure 19:**
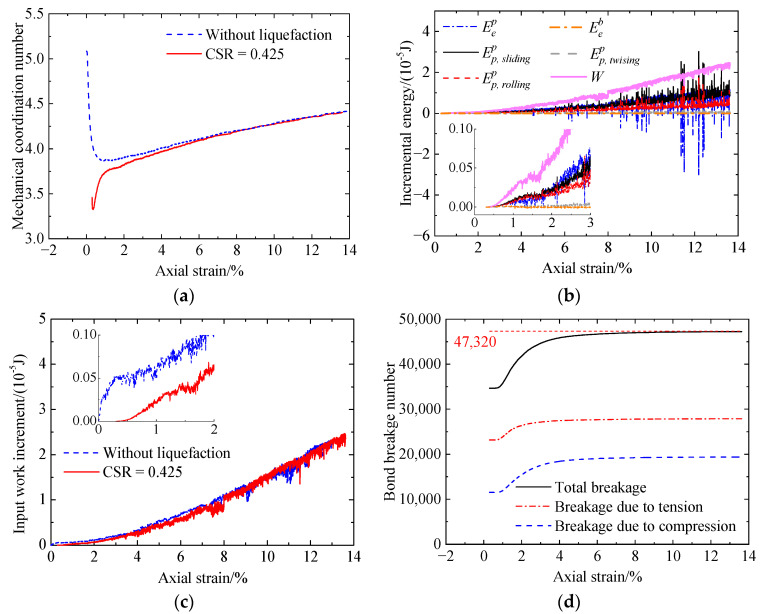
Microscopic monotonic undrained shearing behavior of the cemented sand with CC = 1% after liquefaction under a CSR of 0.425: (**a**) mechanical coordination number versus axial strain; (**b**) energy versus axial strain; (**c**) input work increment versus axial strain; (**d**) bond breakage versus axial strain.

Figure 19 shows the microscopic behavior of the cemented sand with CC = 1% after liquefaction under a CSR of 0.425, including mechanical coordination number ($Z_{m}$) versus axial strain (Figure 19a), incremental energy versus axial strain (Figure 19b), and bond breakage number versus axial strain (Figure 19c). As shown in Figure 19a, the initial $Z_{m}$ is smaller than 3.5 due to a large amount of bond breakage occurring upon cyclic liquefaction (Figure 19c). As a result, the cemented sand exhibits a low initial stiffness (Figure 8). At the initial stage, $Z_{m}$ first increases abruptly at strains smaller than 0.7% and then increases at a decreasing rate. Referring to the bond breakage number, it remains unchanged at strains smaller than 0.7% and then gradually increases at a decreasing rate. Finally, almost all the bonds fail. From the above analysis, the initial sharp increase in $Z_{m}$ is attributed to the increase in the unbonded contacts upon shearing. As loading further proceeds, the increase in the unbonded contacts at a decreasing rate slows the increasing rate of $Z_{m}$. In addition, the main bond breakage type is breakage due to tension, which is different from that for the cemented sand without liquefaction (Figure 12). Figure 19b shows that the incremental elastic energy stored at bond contacts is slight due to a small number of intact bonds. Likewise, the increases in the sliding and rolling energy increments and the incremental elastic energy at particle contacts lead to the increase in the shear strength. Compared with the cemented sand (CC = 1%) without liquefaction, $Z_{m}$ and the input work increment are smaller (Figure 19a,c), indicating a smaller shear strength (Figure 8).

**Figure 20:**
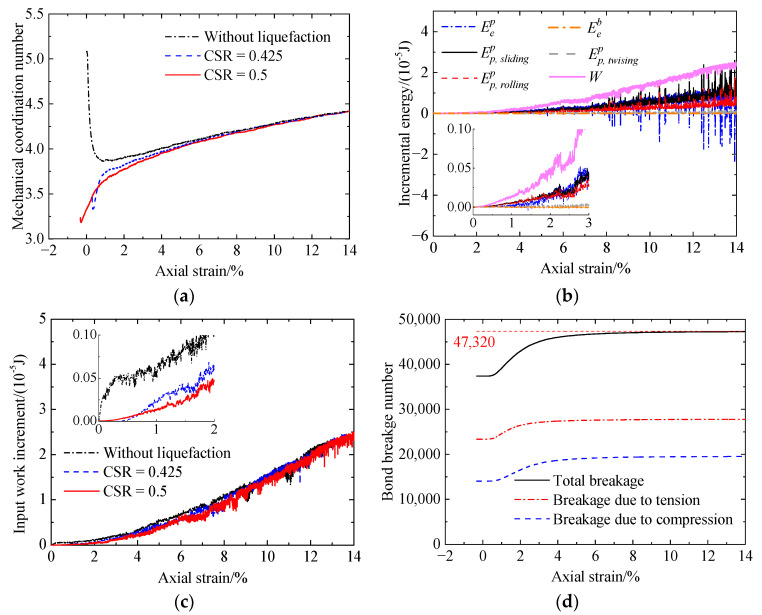
Microscopic monotonic undrained shearing behavior of the cemented sand with CC = 1% after liquefaction under a CSR of 0.5: (**a**) mechanical coordination number versus axial strain; (**b**) energy versus axial strain; (**c**) input work increment versus axial strain; (**d**) bond breakage versus axial strain.

Increasing the CSR from 0.425 to 0.5 (Figure 20), the initial bond breakage number is greater (Figure 19d and Figure 20d). Meanwhile, $Z_{m}$ (Figure 20a) and the input work increment decrease (Figure 20c).

**Figure 21:**
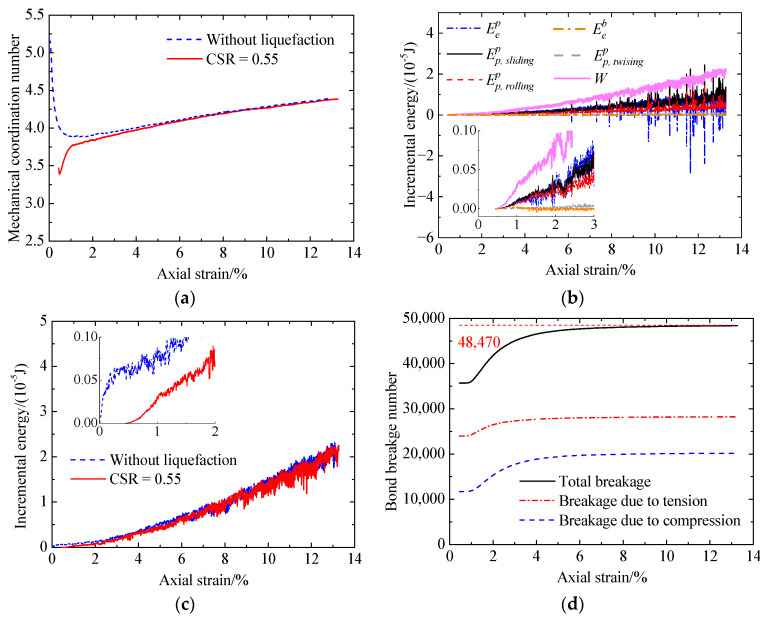
Microscopic post-liquefaction undrained shearing behavior of the cemented sand with CC = 2% after liquefaction under a CSR of 0.55: (**a**) mechanical coordination number versus axial strain; (**b**) energy versus axial strain; (**c**) input work increment versus axial strain; (**d**) bond breakage versus axial strain.

**Figure 22:**
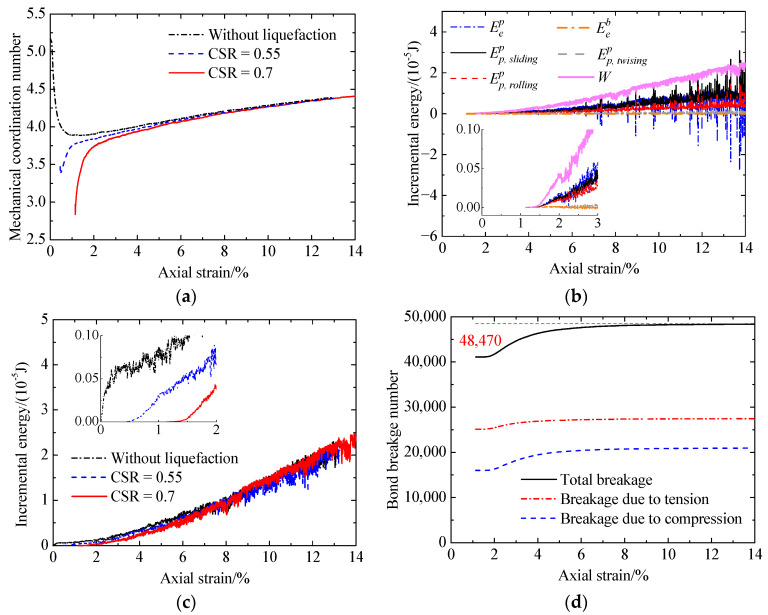
Microscopic monotonic undrained shearing behavior of the cemented sand with CC = 2% after liquefaction under a CSR of 0.7: (**a**) mechanical coordination number versus axial strain; (**b**) energy versus axial strain; (**c**) input work increment versus axial strain; (**d**) bond breakage versus axial strain.

In the case of the cemented sand with CC = 2% (Figure 21 and Figure 22), the evolution of the mechanical coordination number, input work increment, and bond breakage is the same as that of the cemented sand with CC = 1% (Figure 19 and Figure 20). An increase in the CSR leads to an increase in bond breakage (Figure 21d and Figure 22d). Meanwhile, the mechanical coordination number (Figure 22a) and the input work increment (Figure 22c) decrease. Further, prior liquefaction reduces the two microscopic quantities (Figure 22a,c). Consequently, the liquefied specimen has a lower stiffness and shear strength (Figure 9).

## 4. Conclusions

In this study, a series of cyclic undrained triaxial tests followed by post-liquefaction monotonic undrained triaxial tests were simulated using the modified DEM code incorporating a bond contact model, where diverse CCs and CSRs were considered. The post-liquefaction behavior of the cemented sand was studied at the macro- and microscales. The major findings of this study are as follows:

(1) Referring to the specimen without liquefaction, as the CC increases, the dilative behavior becomes more notable, and the shear strength is greater. After experiencing liquefaction, the specimen exhibits a dilative volumetric tendency but possesses a lower initial stiffness and shear strength regardless of CCs and CSRs. For the liquefied uncemented sand, the shear strength increases with CSR, while the opposite is true in the case of the liquefied cemented sand. In addition, the failure envelope is independent of CCs and CSRs. These results are in accordance with the experimental results, indicating the reliability of the DEM simulations.

(2) For the specimen without liquefaction, as loading proceeds, the mechanical coordination number initially drops abruptly and then gradually increases. The evolution of the input work increment is similar to that of the deviator stress, where sliding and rolling energy increments and elastic energy at particle contacts dominate. At the initial stage, bond breakage occurs mainly due to tension, followed by breakage mainly due to compression. The deviator fabric increases at a decreasing rate from zero to a constant value. With increasing CC, the mechanical coordination number and the input work increment increase, while the deviator fabric changes irregularly, leading to a greater initial stiffness and shear strength.

(3) In the case of the liquefied specimen, the mechanical coordination number is initially smaller than 3.5 and then increases to about 4.5 at a decreasing rate, leading to a very low initial stiffness. The initial incremental energy components are smaller than those for the virgin specimen. For the uncemented sand, an increase in the CSR increases the mechanical coordination number and the input work increment. On the contrary, both the mechanical coordination number and the input work increment decrease with an increasing CSR for the cemented sand. Bond breakage due to tension is the main type, which differs from that of the virgin specimen. The evolution of the microscopic quantities controls the effect of cementation and liquefaction on the macroscopic post-liquefaction behavior.

The findings obtained in this study will shed light on understanding the fundamental mechanism underlying the influence of liquefaction and cementation on the mechanical behavior of sand. In addition, the findings will contribute to the establishment of the macro constitutive model for cemented sand experiencing liquefaction, which considers the evolution of microscale quantities. By further using the finite element method incorporating the constitutive model, the design of structures in liquefaction-prone areas or the assessment of soil improvement techniques can be easily made.

It should be noted that the findings in this study are applicable to cemented sand with low levels of cementation. The mechanism underlying the post-liquefaction behavior of cemented sand with high cement contents needs further study, where the cementation-induced densification effect cannot be ignored. To this aim, it is essential to use the bond contact model combined with small grains representing actual cementation agents to simulate cemented sand.

The cyclic liquefaction behavior of cemented sand has been investigated in prior work, where all the microparameters were determined in detail. Therefore, in this study, the same parameters were used. Compared with the experimental data, the DEM model can reflect the influence of cementation and liquefaction on the mechanical behavior of sand. Certainly, the used parameters (e.g., particle stiffness, friction coefficient, among others) will affect the simulation results. In this study, a sensitivity analysis of the parameters was not made.

## Figures and Tables

**Table 1:** Microparameters in DEM simulations [31].

Contact Type	Parameter	Value
Particle contact	Elastic modulus (N/m^2^)	$7.0\times 10^{8}$
	Ratio of the normal stiffness to the tangential stiffness	5
	Shape coefficient	0.25
	Coefficient of local crushing	2.1
	Frictional coefficient	0.5
Bond contact	Elastic modulus (N/m^2^)	$3.5\times 10^{7}$
	Poisson’s ratio	0.32
	Tensile strength (N/m^2^)	$0.5\times 10^{6}$
	Compressive strength (N/m^2^)	$0.5\times 10^{7}$
	Critical slenderness ratio	0.05
	Coefficient of bond radius (CC = 1%) Coefficient of bond radius (CC = 2%)	0.332 0.39
	Elastic modulus (N/m^2^)	$3.5\times 10^{7}$

**Table 2:** Summary of DEM simulation.

DEM Specimens	Cyclic Stress Ratios	Test Stages
CC = 0%	0.125, 0.2	C, M
CC = 0%	0.1, 0.15	C
CC = 0%	-	M
CC = 1%	0.425, 0.5	C, M
CC = 1%	0.2, 0.4, 0.45	C
CC = 1%	-	M
CC = 2%	0.55, 0.7	C, M
CC = 2%	0.5, 0.6, 0.65	C
CC = 2%	-	M

Note: C, cyclic undrained shearing; M, monotonic undrained shearing.

## Data Availability

The original contributions presented in the study are included in the article, further inquiries can be directed to the corresponding author.
